# Effect of cutting tool with micro-pits texture on wood cutting performance

**DOI:** 10.1371/journal.pone.0214888

**Published:** 2019-04-04

**Authors:** Weiguang Li, Zhankuan Zhang

**Affiliations:** 1 Research Institute of Forestry New Technology, Chinese Academy of Forestry, Haidian District, Beijing, China; 2 Research Institute of Wood Industry, Chinese Academy of Forestry, Haidian District, Beijing, China; University of Vigo, SPAIN

## Abstract

A reasonable micro-pits texture has been initially proved that it can improve friction characteristics between wood and cemented carbide surface and reduce surface friction coefficient. In order to study the cutting performance of the micro-texture when it is applied to the cutting tool for cutting wood more effectively, this paper selected micro-pit texture for studying influence of surface micro-texture cutting tool on wood cutting performance and cutting temperature, finding that when micro-pit cemented carbide cutting tool is adopted for turning the northeast China ash (*Fraxinus* spp.), it can reduce cutting force of turning and surface friction coefficient between rake face and cuttings. Moreover, for type A and type B cutting tools, when the texture parameters are that the diameter of the micro-pit is 80μm, the depth of the micro pit is: 10μm, area occupancy is 20% and the diameter of the micro-pit is 120μm, the depth of the micro-pit is 10μm and the area occupancy is 20%, the effect generated is the best. When a texture cutting tool is used for cutting, the decrease of the highest temperature in the cutting area is not very great, but the average temperature in the cutting area changes a lot, which is mainly caused by that micro-texture is processed at a position of the rake face close to the main cutting edge and that the highest temperature of cutting is mainly generated on the contact point between tool tip and wood. A reasonable micro-texture parameter can form a layer of liquid lubricating film on the up and down contact surfaces such that the direct contact between cemented carbide and northeast China ash is changed into indirect contact between lubricating films formed by the liquid so as to reduce the surface friction coefficient.

## Introduction

Friction plays an important role in the wood cutting process. A large part of the energy consumed in wood cutting is used to overcome the friction generated by wood workpiece and tool surface [1]. Especially, when considering the direct effect of the tool rake face and the chips formed, it is quite necessary to correctly understand the friction phenomenon. The friction force acting on the tool rake surface not only directly affects the tool wear, but also affects the surface quality of the machined workpiece[2–4], so reducing the coefficient of friction between the cutting tool and the wood in the wood cutting process has always been a core issue in the field of wood cutting.

Mckenzie and Karpovich[5] were the first to analyze the elastic-plastic model of friction during wood cutting. Guan[6] studied the relationship between friction coefficients of wood and metal when the moisture content of wood is low (≤30%) and when the moisture content of wood is high (≤40%-50%). McMillin[7] and Murase[8,9]studied the influence of free water in wood on the friction coefficient of wood surface systematically. They argued that the reduction of friction coefficient by moisture lubrication is affected by the initial moisture content of wood, wood section and wood species. The other studies of contact between wood and metal have examined conditions, such as the friction of wood and metal under high speed[10], the effect of wood flowability when forming wood with a metal under high pressure, and pressing wood veneer with heated plates[11].

Currently, cemented carbide is the most widely used material in woodworking tools. Super-hard ceramics [12], coating technologies[13,14], and the optimization of the cutting tool structure[15] are commonly used to reduce the coefficient of friction. Micro-textures can improve material friction performance[16,17] and they have been applied in friction between the wood and metal[18], and mainly considering the effect of moisture content of wood and loading on friction coefficient. However, all the above studies are based on the analysis of the friction test of the face-to-face friction pair on the friction and wear testing machine, In order to study the cutting performance of the micro-texture when it is applied to the wood cutting more effectively, this paper selected micro-pit texture for studying influence of surface micro-texture cutting tool on wood cutting force and cutting temperature, so as to provide a basis for the design of reasonable wood cutting tool.

## Materials and methods

### Wood materials

The green wood samples from the northeast China ash (*Fraxinus* spp.) derived from the northeast region in China and used in the experiments, with an average moisture content of 67% and an average air-dry density of 0.72 g/cm^3^, which adopted as wood specimen and it was processed through rotary cut to form a cylinder-shaped specimen having a diameter of 100 mm and a length of 1000 mm.

### Cutting tools

For the experiment, the cemented carbide turning tool is made up of YG8 (WC:92%,Co:8%), which had an density of 14.7g/cm^3^, heat conduction coefficient of 75.4 W/m·K, flexure strength of 1500MPa and hardness of 89 HRA. The cutting tools had 18° clearance angle, 42° cutting edge angle, and the tool cutting edge inclination is 0°. The edge radius of the cutting tool edge is 0.4mm and surface roughness parameter Ra of the rake face of the cutting tool is 0.4μm. In order to reflect the friction effect between the rake face and the chip better, the research adopted a cutting tool having a rake angle of 0° for cutting experiment.

**Fig 1:**
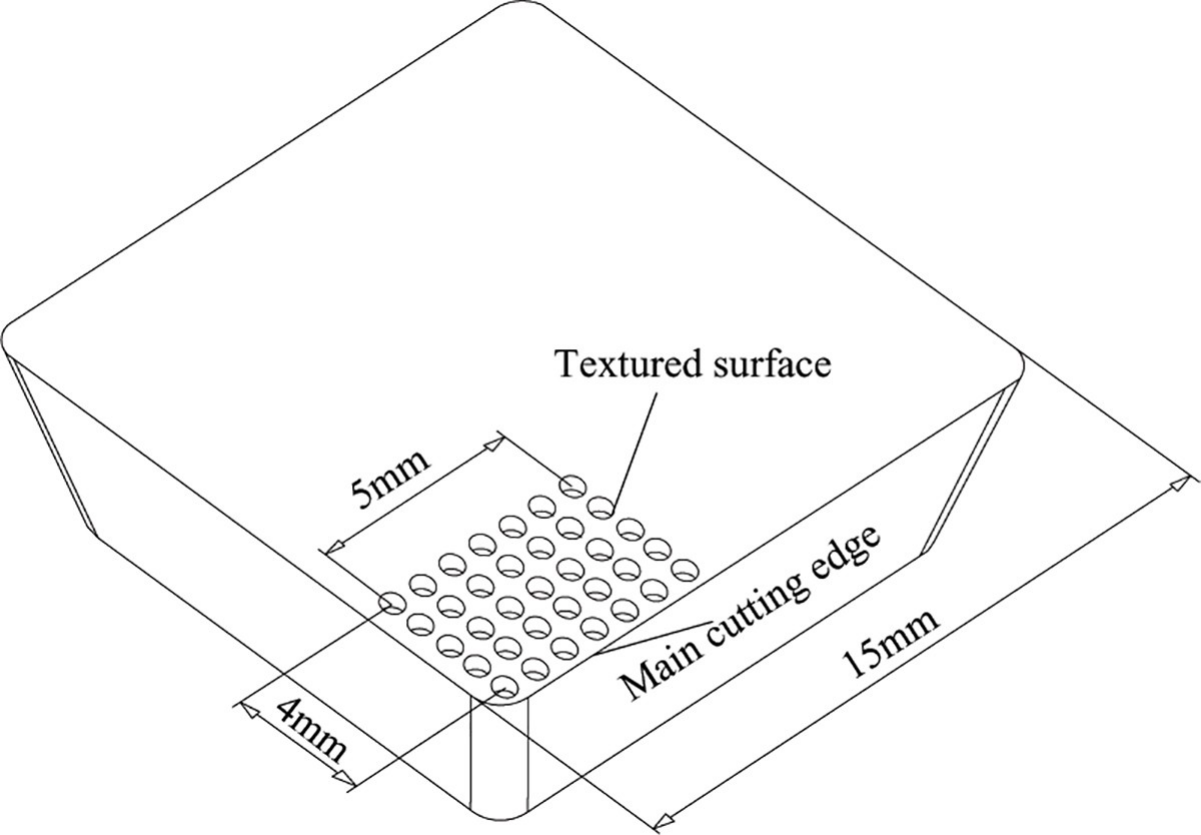
Diagram of micro-pit texture cutting tool.

**Fig 2:**
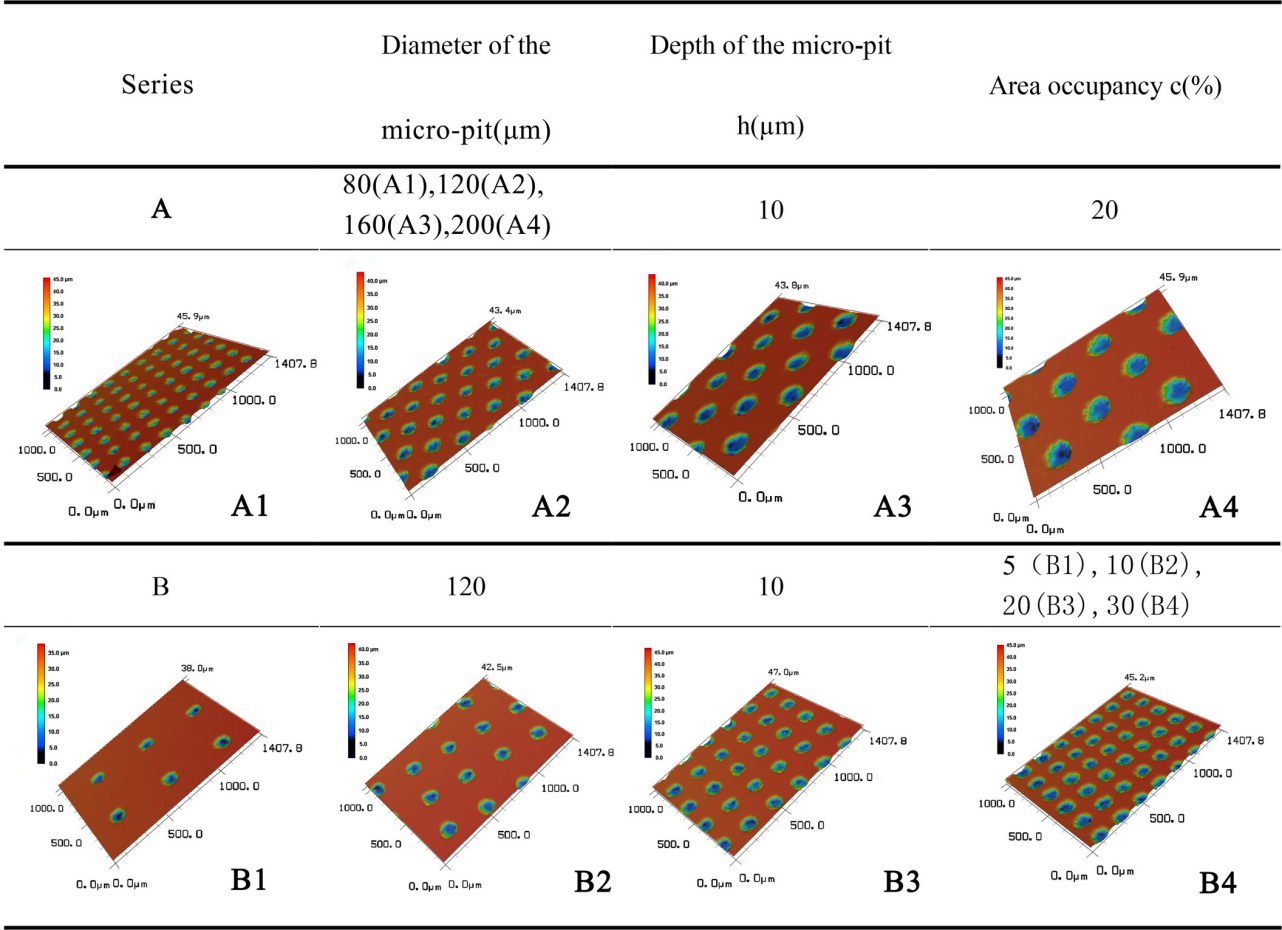
Parameters of the experiment.

As shown in Fig 1, micro-texture infrared processing is performed on the rake face close to the main cutting edge. The type of the texture is micro-pit type. Processing type of textured cutting tool can be divided into two groups: A and B (four specimen/one group). Parameters and numerals of morphology to be processed in each group are shown in Fig 2. A series of cutting tools are used to study the effect of micro-pit diameter on cutting characteristics under the same area occupancy (20%) and micro-pit depth (10 μm), and B series of cutting tools are used to study the effect of area occupancy on cutting characteristics under the same micro-pit diameter (120μm) and micro-pit depth (10 μm). In order to remove burr generated around the micro-pit in the infrared processing, abrasive paper(#1000) for metallograph was used for removing burr. Later, acetone was taken as cleaning fluid for cleaning specimens using ultrasonic cleaner.

### Experiment device and method

**Fig 3:**
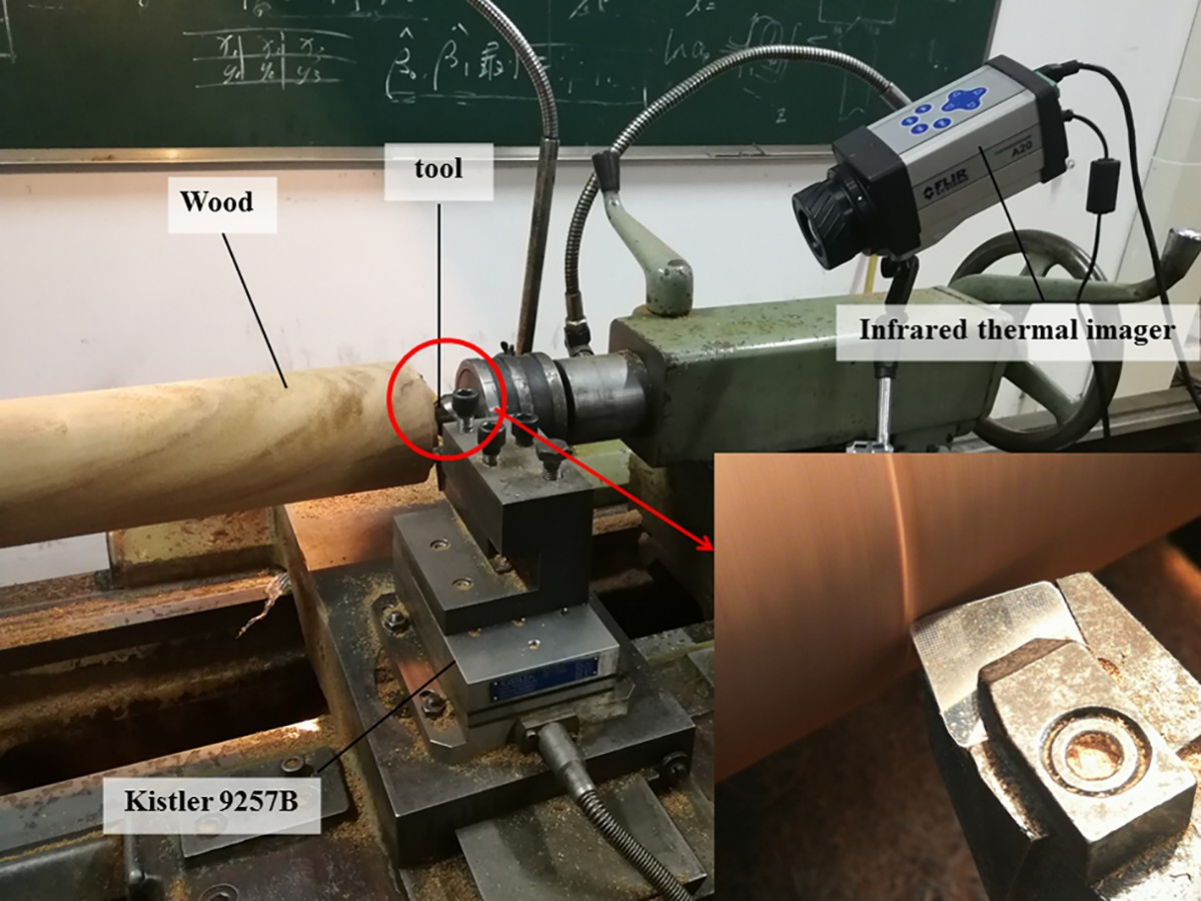
Cutting experiment device.

**Fig 4:**
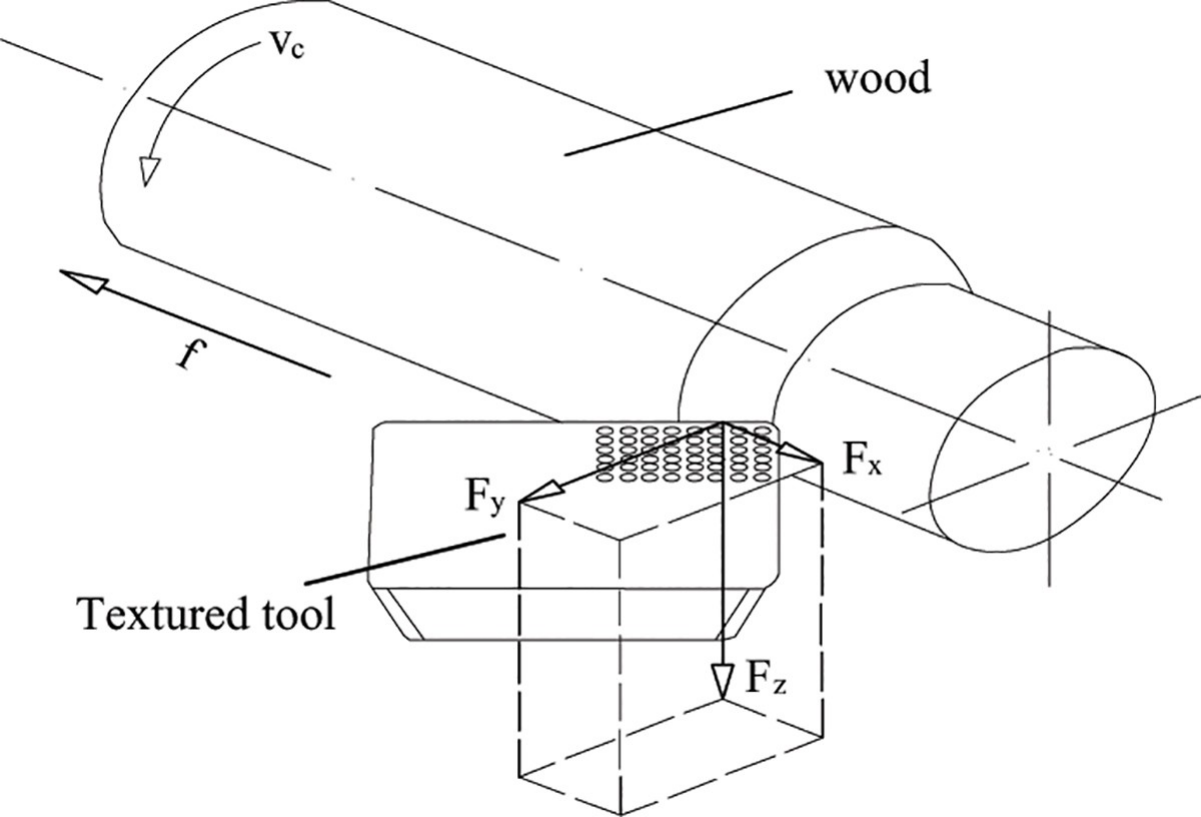
Diagram on cutting of a micro-texture cutting tool.

**Fig 5:**
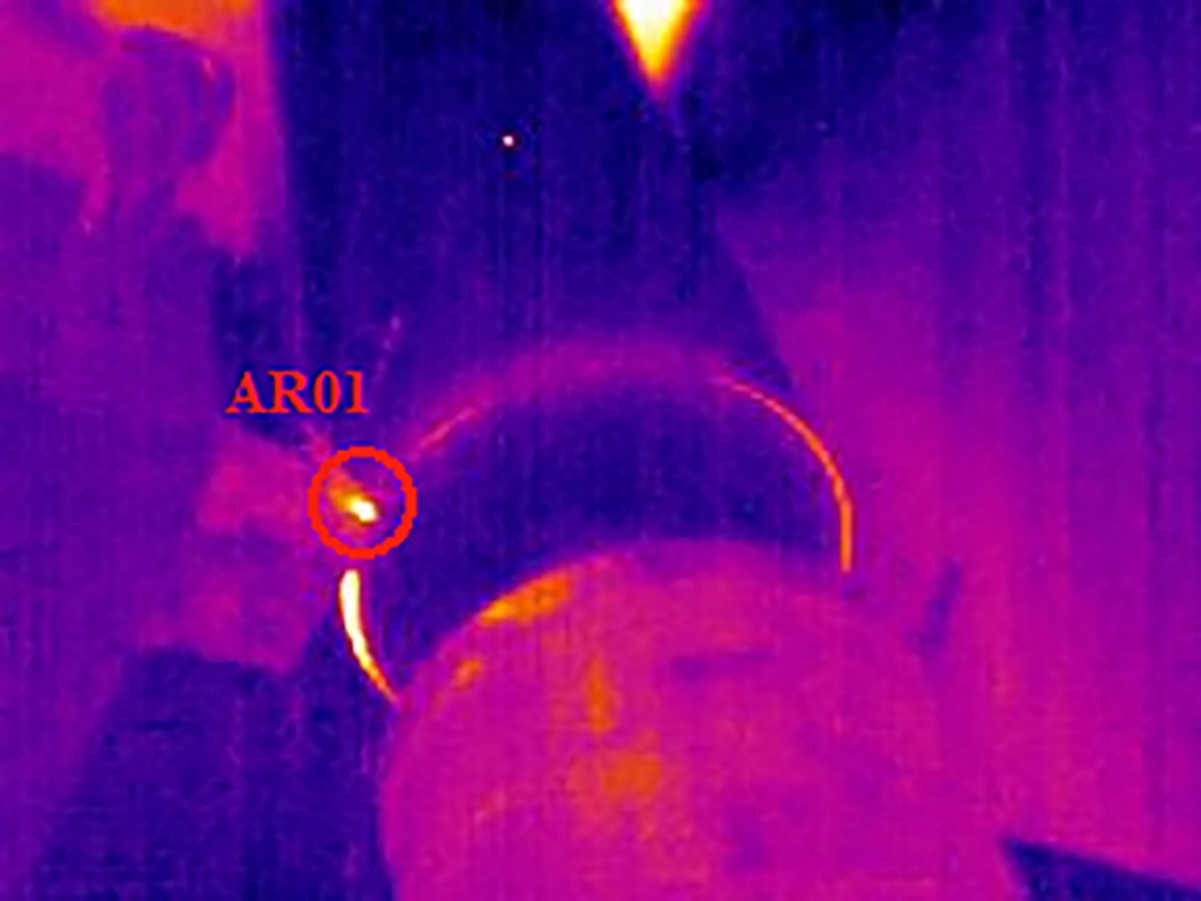
Infrared thermal image of cutting tool area.

The environment temperature was 23°C and the relative humidity was 55%. Textured cutting tools and non-texture tool were used to perform wood turning experiment on the cutting experiment table transformed from CA6140 turning machine, as shown in Fig 3. By using the Kistler (9257B type) three-dimensional dynamometer, the axial force Fx, the normal force Fy and the main cutting force Fz were measured as shown in Fig 4. In the test, the sampling frequency was 3.0×10^3^ Hz. According to geometric parameters and wood turning theory of the cutting tool, Eq (1) to Eq (8) can be obtained. By combining with the data of cutting force in each direction, the resultant force of cutting F and the friction coefficient between the rake face and cuttings can be calculated. Under the same cutting condition, the experiment adopted single-factor and multi-level experiment design scheme to perform cutting experiment over the texture cutting tool and the ordinary cutting tool, compare influence of micro-pit surface texture on cutting force of the cutting tool and surface friction characteristics, and measure changes of temperature field for comparison when different forms of texture cutting tools and ordinary cutting tools are used for cutting by adopting FILR infrared thermal imager (A20 type). The distance between the thermal imager and the tip of the tool is 40 cm, the sampling frequency was 50 Hz. With infrared thermal image temperature field of cutting shown in Fig 5, Thermal CAM Researcher Pro 2.7 analysis software of the infrared thermal imager was adopted for comparing maximum temperature T_Max_ and average temperature T_Avg_ of cutting in AR01 area in cutting process by setting a round area AR01 having a tool tip as the center and a radius of 1cm as the temperature field of the cutting area. The experiment design is shown in Table 1. In order to minimize the inaccuracies caused by the tool wear and other factors, different types of tools used in the study have the same edge radius of the cutting tool edge, 3 cuts were made by the same type of cutting tool in each test, a total of 54 cuts were made according to the experimental design in this study. Each cutting time is 7 seconds, recording the force and temperature of each test, and average value is selected as the experimental determination value.

**Fig 6:**
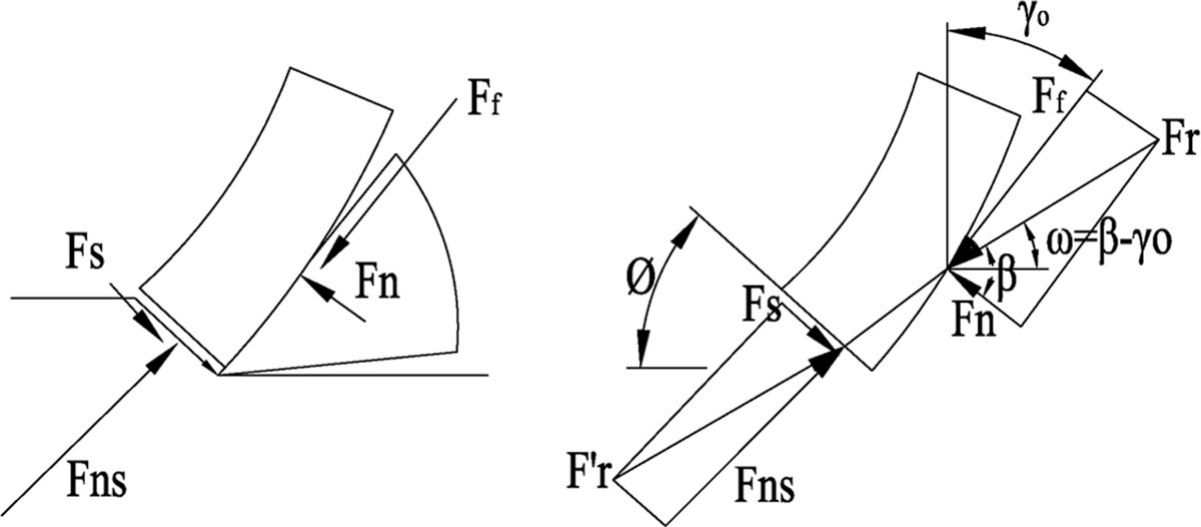
The force acting on the chip.

**Fig 7:**
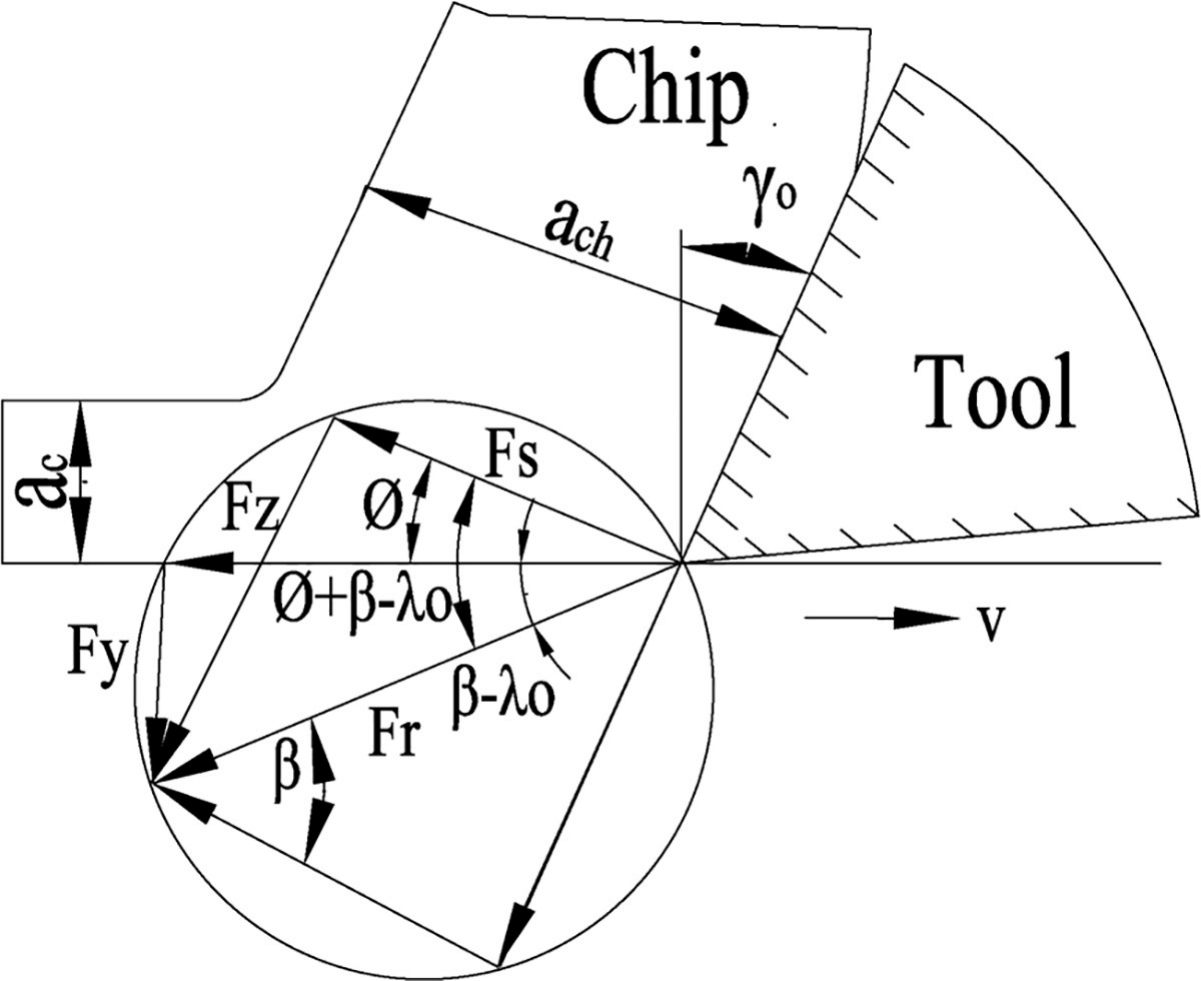
Relationship between cutting force and angle.

The forces acting on the chip during cutting are normal force F_n_ and frictional force F_f_ on the rake face, there is also a positive pressure F_ns_ and shear force F_s_ on the shear plane, as shown in Fig 6. The two pairs of forces should balance each other. If all the force is drawn in front of the cutting edge, the relationships of the forces are shown in Fig 7. F_r_ is the resultant of F_n_ and F_f_, also known as chip forming force; ϕ is the shear angle; β is the angle between F_n_ and F_r_, also known as the angle of friction; γ_0_ is the rake angle; F_z_ is the component of the cutting motion direction, F_y_ is the cutting component perpendicular to the direction of cutting motion, a_c_ is the cutting thickness.

The cutting width is indicated by a_w_ and the sectional area of the cutting layer is indicated by A_c_ (*A_c_* = *a_c_a_w_*), A_s_ represents the sectional area of the shear plane $\left(A_{S}=\frac{A_{C}}{\sin\;\emptyset }\right)$, τ represents the shear stress of the shear plane, then:

$$F_{S}=\tau A_{S}=\frac{\tau A_{c}}{\sin\;\emptyset }$$ (1)

$$F_{S}=F_{r}\;\cos\left(\emptyset +\beta -\gamma_{0}\right)$$ (2)

$$F_{r}=\frac{F_{S}}{cos(\emptyset +\beta -\gamma_{0})}=\frac{\tau A_{c}}{\sin\;\emptyset \;\cos\left(\emptyset +\beta -\gamma_{0}\right)}$$ (3)

$$F_{z}=F_{r}\;\cos(\beta -\gamma_{0})=\frac{\tau A_{c}\;\cos\left(\beta -\gamma_{0}\right)}{\sin\;\emptyset \;\cos\left(\emptyset +\beta -\gamma_{0}\right)}$$ (4)

$$F_{y}=F_{r}\;\sin\left(\beta -\gamma_{0}\right)=\frac{\tau A_{c}\;\sin\left(\beta -\gamma_{0}\right)}{\sin\;\emptyset \;\cos\left(\emptyset +\beta -\gamma_{0}\right)}$$ (5)

If the values of F_z_ and F_y_ are measured by force meter and ignore the force on the clearance face:

$$\frac{F_{y}}{F_{Z}}=\tan\left(\beta -\gamma_{0}\right)$$ (6)

tanβ is the average friction coefficient on the rake face, when the rake angle is 0°, it can be known:

$$\mu =\tan\;\beta =\frac{F_{y}}{F_{Z}}$$ (7)

The resultant force of cutting F is:

$$F=\sqrt{F_{x}^{2}+F_{y}^{2}+F_{Z}^{2}}$$ (8)

According to wood turning theory, Fx is axial force, Fy is radial force, Fz is main cutting force.

**Table 1:** Design table of cutting experiment.

Factors	Parameters
Species	Northeast China ash
Type of cutting tool	A1, A2, A3, A4, B1, B2, B3, B4, Non-textured tool
Cutting depth	0.8 mm
Cutting speed	290 m/min
Feed rate	0.51 mm/r
Angle between edge and wood fiber	45°
Test value 1	Fx: axial force/N; Fy: normal force/N; Fz: main cutting force
Test value 2	Maximum temperature T_Max_; Average temperature T_Avg_

## Results and discussion

**Fig 8:**
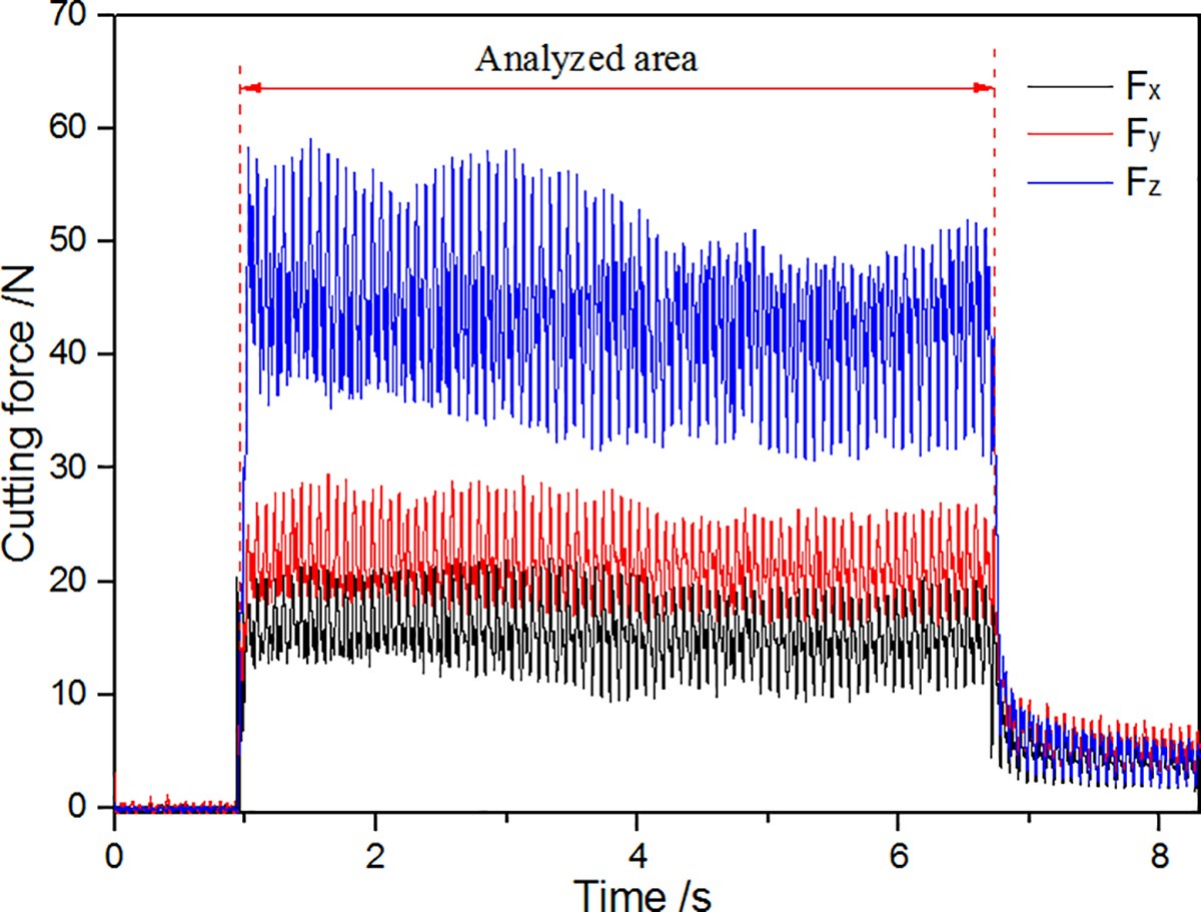
Data of cutting force.

**Fig 9:**
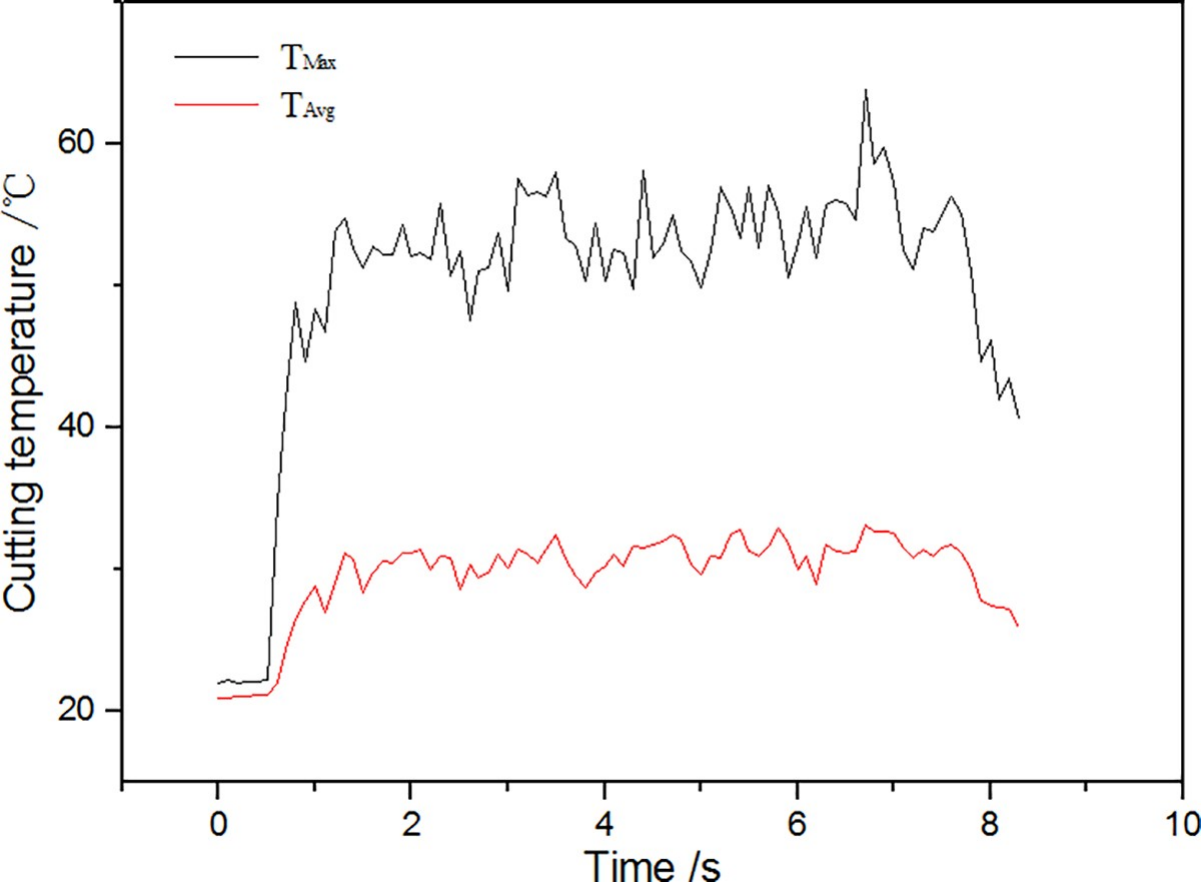
Curve on changes of the temperature in AR01.

**Fig 10:**
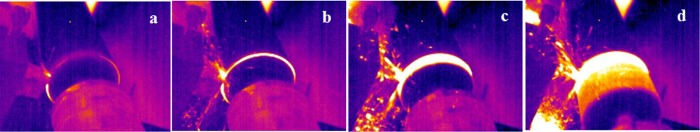
**Thermal infrared diagram of temperature field in the cutting area:** a. 0.1s, T_Max_:34.7°C; b. 2s T_Max_:52.1°C; c. 4s, T_Max_:54.4°C; d. 7s, T_Max_:63.8°C.

Fig 8 shows the changes of cutting force using Kistler measuring for analyzing signal fitting of software DynoWare. As shown in Fig 8, the main cutting force (Fz) > the normal force (Fy)> the axial force (Fx) using a cutting tool with zero rake angle. Fig 9 shows the curve about changes of the highest temperature and average temperature in cutting area(AR01) when the non-texture cutting tool analyzed by using Thermal CAM Researcher Pro 2.7 analysis software of infrared thermal imager is used for turning ash. As shown in Fig 9, the highest temperature of cutting in the cutting area is 63.8°C and the average temperature reaches a highness of 33.2°C. Fig 10 shows the thermal infrared diagram of a temperature field in cutting area at a different moment using non textured tools.

### Effects of the mico-texture on cutting force

**Fig 11:**
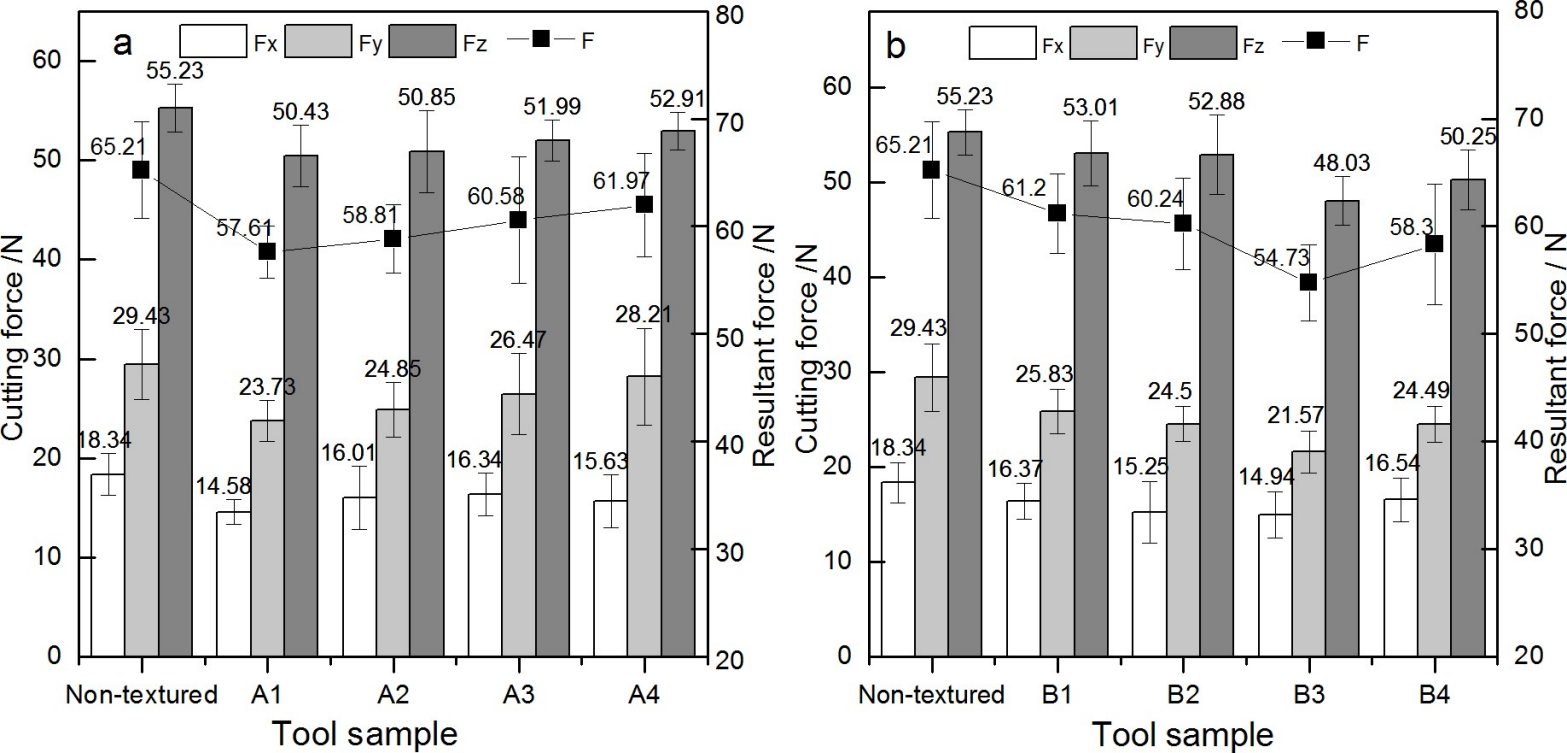
**Influence of a different type cutting tool on cutting force:** a. A series; b. B series.

Fig 11 shows the influence of non-textured tool and textured tools on cutting force and resultant force. As shown in the Fig 11, the main cutting force Fz is greater than axial force Fx and radial force Fy. No matter A series or B series, cutting force in each directions and cutting resultant force generated by textured tools were both smaller than those of non-textured cutting force. For A series of textured cutting tool, as shown in Fig 11A, the axial force, radial force and main cutting force generated by A1 type cutting were smaller than those of other A type tools, and the decrease was the largest. Compared with non-textured cutting tool, the decrease of its axial force was 20.6% (from 18.34N to 14.56N), the decrease of its radial force 19.4% (from 29.43N to 23.73N) and the decrease of its main cutting force 8.7% (from 55.23N to 50.43N). The cutting resultant force decreased by 11.6% (from 65.21N to 57.61N). This shows that for A1 type tool, when the diameter of the texture micro-pit is 80μm, the depth of the micro-pit is 10μm and area occupancy is 20%, each cutting force and resultant force of cutting generated is the smallest. For B series of textured cutting tool, as shown in Fig 11B, when B3 type cutting tool was used for cutting, the axial force, radial force and main cutting force were all smaller than those of other B type cutting tools. Compared with non-textured cutting tool, its axial force, radial force and main cutting force are respectively decreased by 18.5%, 26.7% and 13.0%. The decrease of the resultant force of cutting was 16.1%. That is, when the diameter of the micro-pit of the texture is 120μm, the depth of the micro-pit is 10μm and area occupancy is 20%, each cutting force and resultant force of cutting generated is the smallest. In addition, it can be known from the above that the surface micro-texture has a function of weakening the axial force, radial force, main cutting force and resultant force in the cutting process, which is mainly due to the fact that the cutting forces in all directions need to overcome the friction between the rake face of the cutting tool and the chip. The friction between metal and wood is partly due to the close contact between the two specimen when there is a certain pressure. When the contact is close enough and the contact gap is smaller than the molecular attraction radius, the intermolecular force, namely VDW force, will be generated. In addition, due to the hydrogen bond formed between the hydroxyl group of wood material and the oxide layer on the metal surface of the tool, the resultant force of the two forces will hinder the relative movement of the two surfaces to a certain extent. A reasonable micro-pit texture reduces cohesive contact area between the rake face of the cutting tool and chip so as to reduce the cutting force.

### Effects of the mico-texture on friction coefficient between the rake face and chip

**Fig 12:**
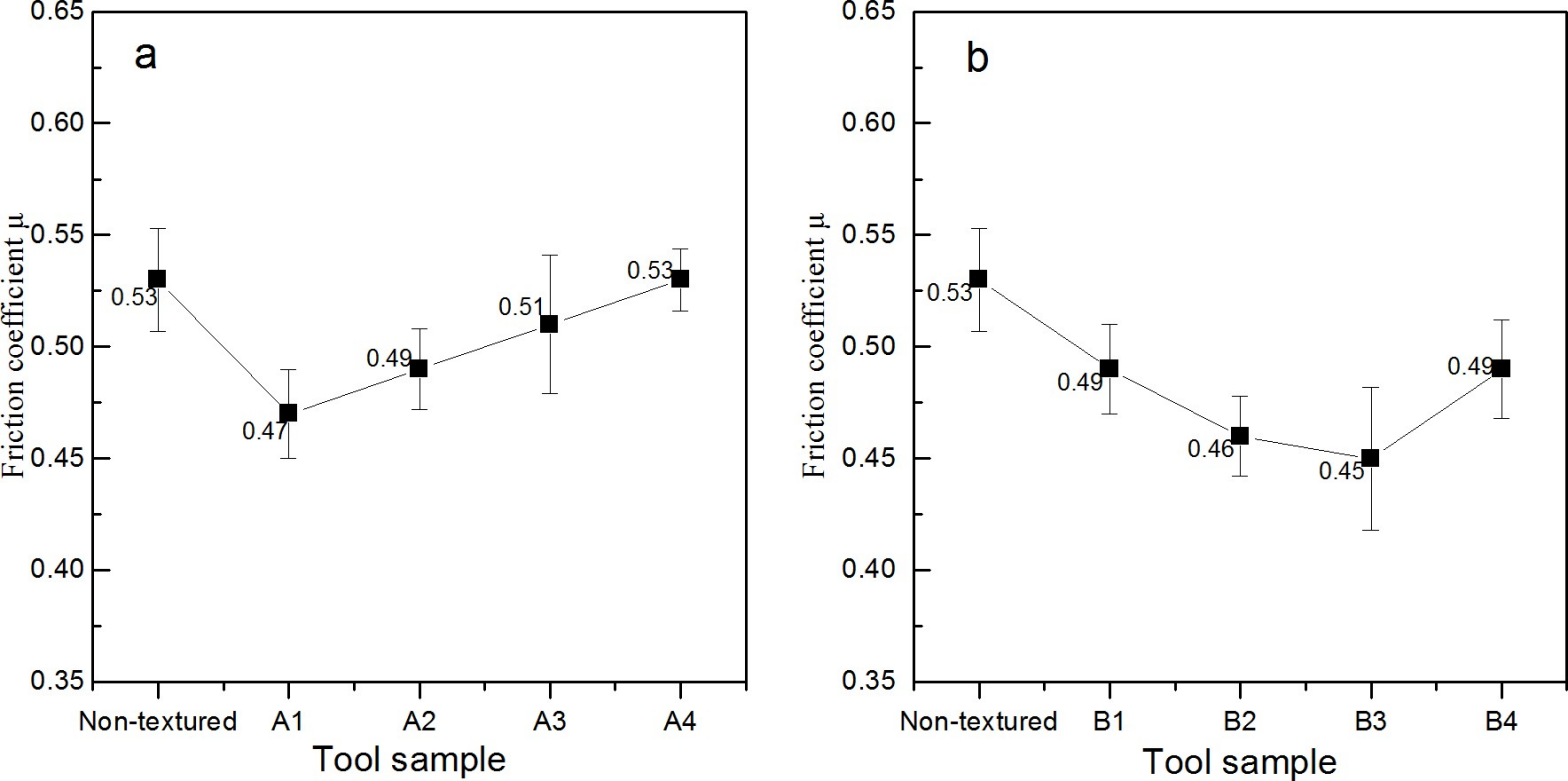
**Influence of different type cutting tools on friction coefficient between the rake face and chips:** a. A series; b. B series.

**Fig 13:**
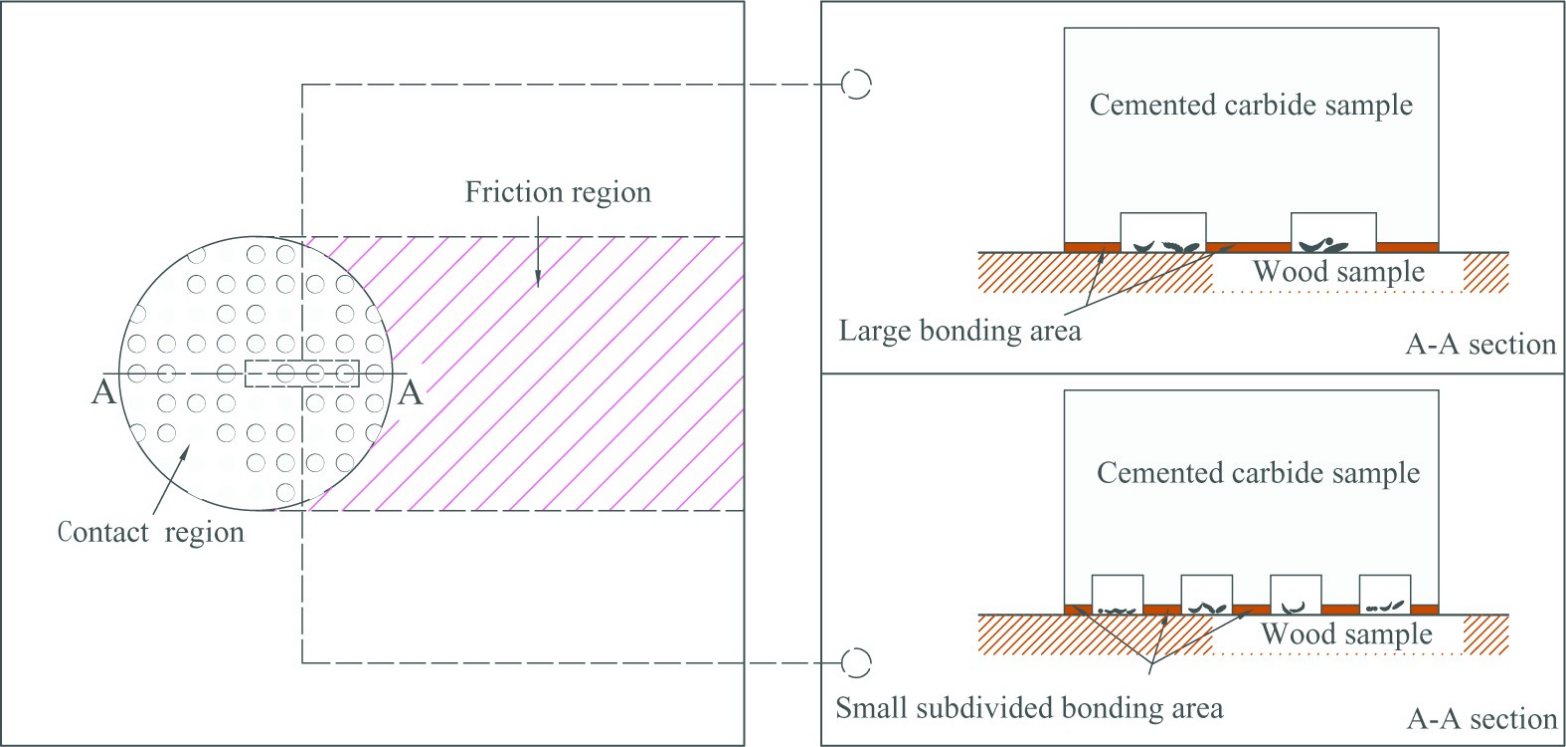
Adhesion of micro-pit texture in friction contact area at the same area ratio.

Fig 12 shows the influence of different type cutting tools on friction coefficient between the rake face and chips. For A series, as shown in Fig 12A, when the diameter of the texture micro-pit is 80μm, the depth of the micro-pit is 10μm, and area occupancy is 20%, the friction coefficient between the rake face of the cutting tool and wood chips was the smallest, reaching 0.47. Moreover, when the diameter of the micro-pit is greater than 80μm, the surface friction coefficient increases with the increase of the diameter of the micro-pit, when the diameter of the pit is 200 μm, the maximum friction coefficient is 0.53. That is, the mico-textured tools with the same area occupancy but smaller pit diameter shows better friction reduction effect. This may be due to that when micro-texture exists, the texture surface with a pit diameter of 80 μ m is "broken" by micro-pits to reduce the contact area between the two specimen and reduce the bonding effect, thus the texture specimen has a lower friction coefficient than the non-texture specimen within a certain diameter range. However, the increase of surface friction coefficient with the increase of pit diameter may be because of the fact that there are more micro-pit structure contained in the micro-texture surface with smaller diameter under the same area ratio of the same size specimen. These micro-pit structures subdivide the contact area so that the adhesion between the two surfaces cannot form a continuous resultant force, as shown in Fig 13.

**Fig 14:**
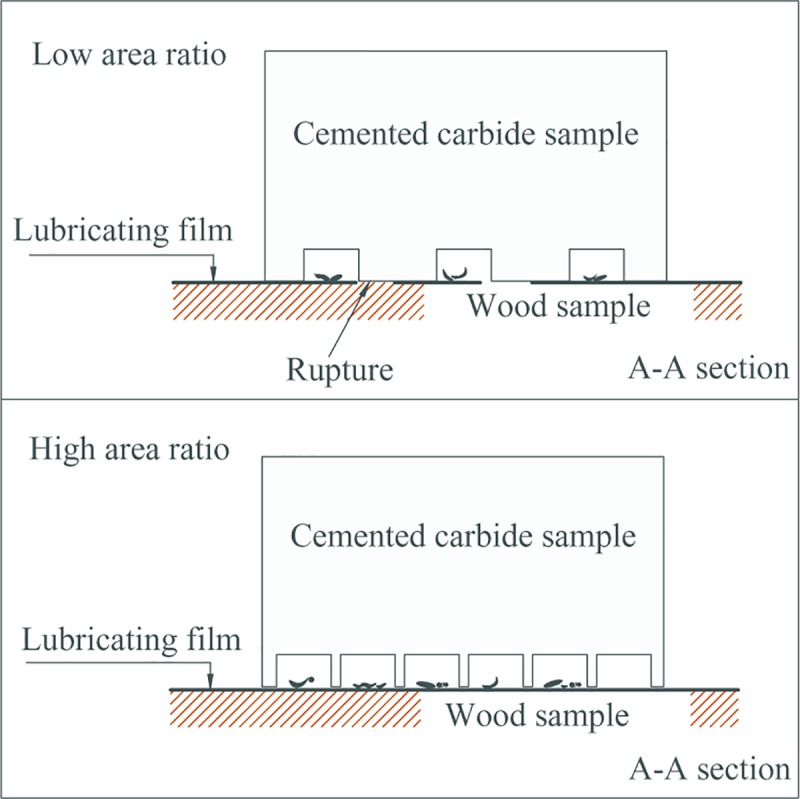
Surface condition of micro texture with different pit area ratio.

For B series, as shown in Fig 12B, the friction coefficient decreases with the increase of area occupancy. When the diameter of the micro-pit is 120μm, the depth of the micro-pit is 10μm, and the area occupancy is 20%, the surface friction coefficient generated on the rake face of the cutting tool and wood chip is the smallest, reaching 0.45. After that, the friction coefficient increases to 0.49. This may be due to that when the area ratio of micro-pits is low, the contact area between wood and cemented carbide is large, and the bonding effect caused by contact pressure is strong. In addition, as the friction progresses, the lubricating film formed on the contact surface is more likely to be damaged to form a plurality of independent lubricating areas, which does not completely cover the contact area, resulting in a larger friction coefficient. However, with the increase of the area ratio of the micro-pits, the contact area between the two surfaces decreases and the bonding effect due to contact pressure decreases, resulting in a decrease in the surface friction coefficient of the wood with two different moisture contents. With the increase of the area ratio, especially for the green specimen, the ability to store moisture in the micro-pits increases, contributing to the formation of a lubricating film on the sliding surface, repairing and supplementing the damaged film, and reducing the surface friction coefficient. However, when the area occupation ratio increases to a certain extent, the surface friction coefficient increases. This is mainly because that with the increase of the texture occupation ratio, the surface roughness of the cemented carbide sample increases, and the bearing area of the sample surface decreases, resulting in the increase of the surface friction coefficient, as shown in Fig 14.

### Effects of the mico-texture on the cutting temperature

**Fig 15:**
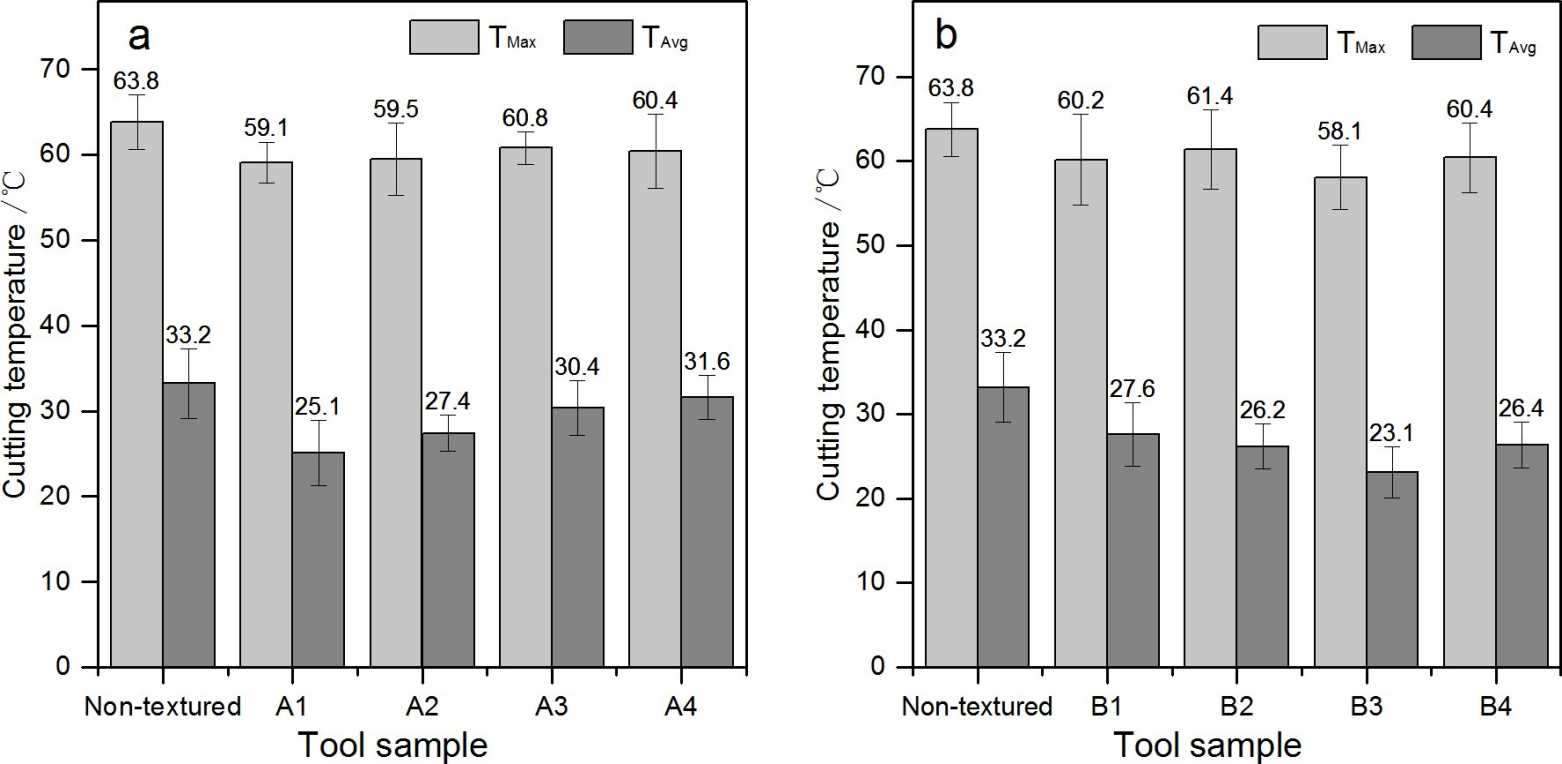
**Influence of different type cutting tools on cutting temperature of the cutting area:** a. A series; b. B series.

Fig 15 shows the influence different type cutting tools on cutting temperature of the cutting area. For A series, as shown in Fig 15A, the decrease of the highest temperature (T_Max_) in the cutting area is not very great, reaching 7.4% (A1, from 63.8°C to 59.1°C), 6.7% (A2, from 63.8°C to 59.5°C), 4.7%(A3, from 63.8°C to 60.8°C) and 5.3% (A4, from 63.8°C to 60.4°C), respectively, when using A type cutting tools. But the average temperature (T_Avg_) in the cutting area changes a lot, and its decrease reaches 24.3% (A1, from 33.2°C to 25.1°C), 17.5% (A2, from 33.2°C to 27.4°C), 8.4% (A3, from 33.2°C to 30.4°C) and 16.9% (A4, from 33.2°C to 31.6°C), respectively. Thus, it can be known that the decrease of the highest temperature and average temperature of A1 type cutting tool in the cutting area is the greatest.

**Fig 16:**
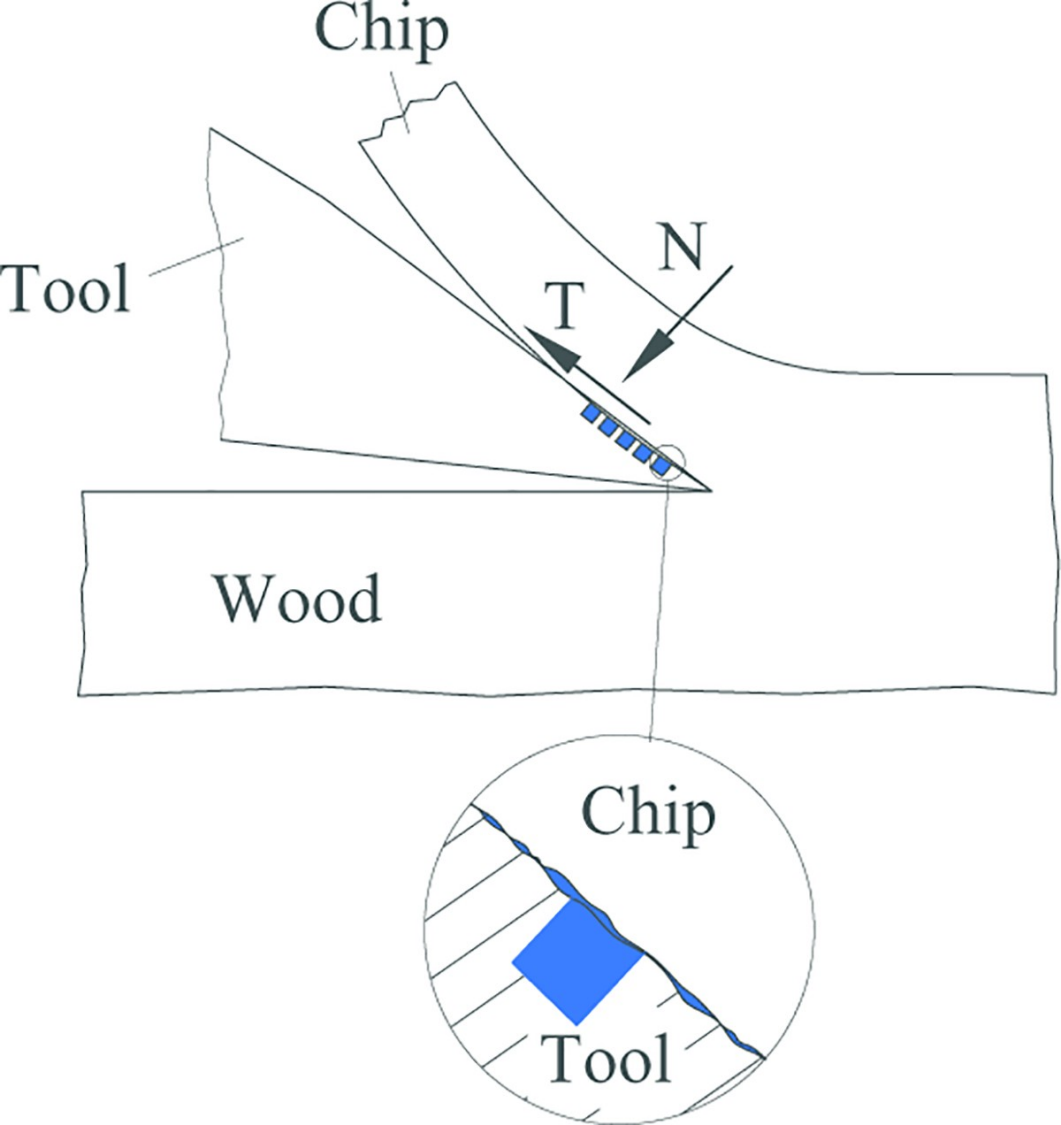
Contact between the rake face of the cutting tool and chip.

**Fig 17:**
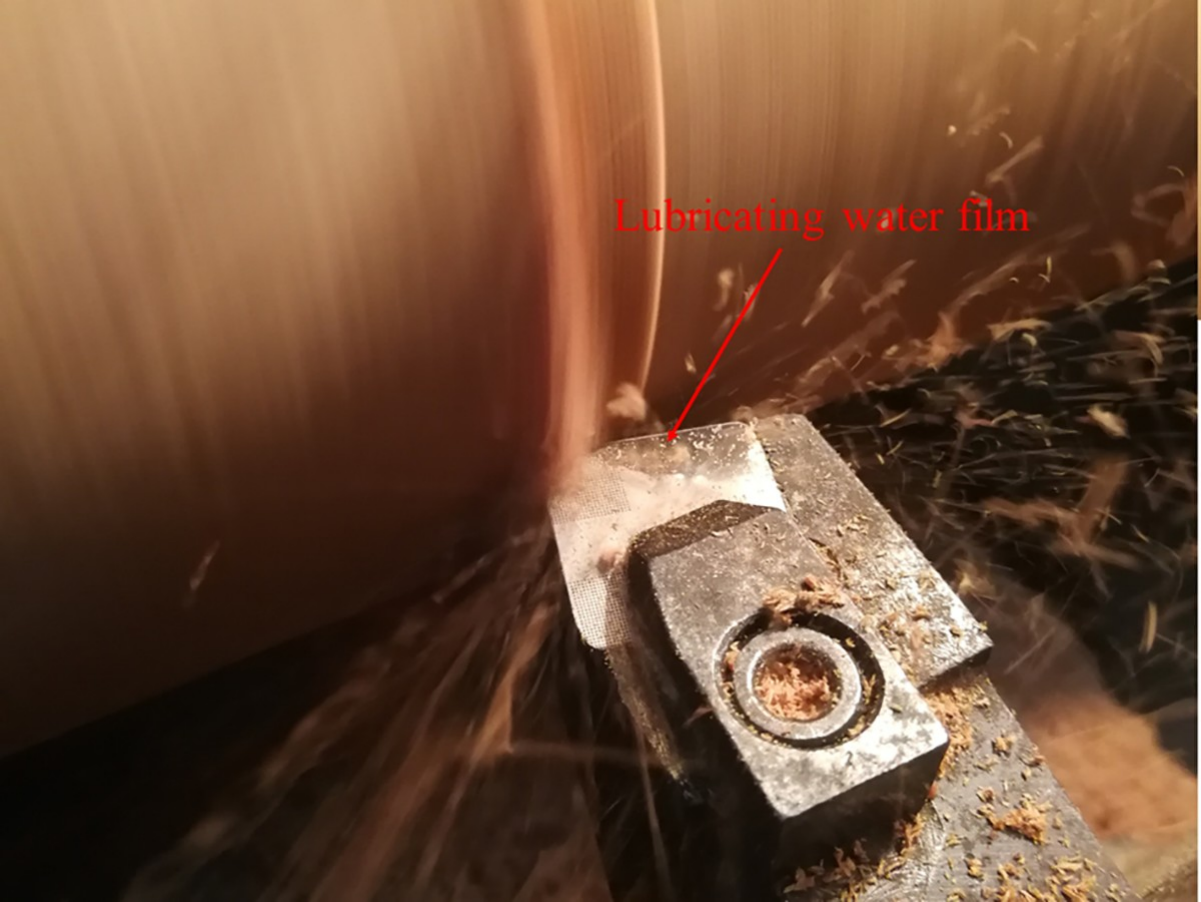
Forming lubricating film with seeped free water.

For B series, as shown in Fig 15B, the change of the highest temperature and average temperature in the cutting area is of the same trend, that is the decrease of the highest temperature is not great, but the decrease of the average temperature is great. The greatest decrease happens when B3 type cutting tool is used with the diameter of the micro-pit is 120μm, the depth of the micro-pit is 10μm, and area occupancy is 20%, the decease of the highest temperature and the decrease of the average temperature is 8.9% and 30.4%, respectively. The main reason for the above phenomenon may be due to that the highest cutting temperature in cutting is mainly generated on the tool tip position in contact with wood. The micro-texture is located on the front cutting edge near the main cutting edge, and as the chip moves along the rake, the interactive action of the wood and the surface of the cutting tool will generate friction force (F) and the positive pressure (N) which vertical to friction force (F), as shown in Fig 16. Suppose that the edge of tool is absolutely sharp, and the flank does not contact a workpiece; in wood cutting process, chips will generate great pressure on the rake face and friction phenomenon. When the mico-texture exists on the rake face of cutting tools, the friction area is reduced. When the micro-pit texture exists, due to extrusion of wood in a cutting layer by cutting edge, cause the free water in the green wood of ash permeate out and a layer of lubricating film is formed on the rake face which is beneficial to reduce friction coefficient between the rake face and chips so as to reduce the cutting temperature in the cutting area, as shown in Fig 17.

## Conclusion

It can reduce the cutting force and the friction coefficient between the rake face and chips when using micro-pit cemented carbide cutting tool for turning the northeast China ash. Moreover, for type A and type B cutting tools, when the texture parameters are: the diameter of the micro-pit is 80μm, the depth of the micro pit is: 10μm, area occupancy is 20% and the diameter of the micro-pit is 120μm, the depth of the micro-pit is 10μm and the area occupancy is 20%, respectively, the effect generated is the best.

Compared with non-textured cutting tool, the decrease of the highest temperature in the cutting area is not very great, but average temperature in the cutting area changes a lot when using micro-pit cemented carbide cutting tool, which is mainly due to that the micro-texture is processed at a position of the rake face close to the main cutting edge and the highest cutting temperature is mainly produced on the tool tip position in contact with wood.

A reasonable micro-texture parameter can effectively reduce the friction coefficient between the northeast China ash and cemented carbide surface. The presence of the micro-texture can reduce the cohesive action of two contact surfaces. In particular for wood having a high moisture content, it can also form a layer of liquid lubricating film on the upper and lower contact surfaces, so that the direct contact between cemented carbide and wood can be changed into indirect contact between the lubricating film formed by liquid, so as to reduce the surface friction coefficient.
